# Incidence, Management, and Hospital Costs of Orthopaedic Injuries of E-Scooter Riders in Western Australia

**DOI:** 10.3390/jcm12206591

**Published:** 2023-10-18

**Authors:** Kyle Raubenheimer, Katherine Szeliga, Jonathan R. Manara, Daniel M. Fatovich, James G. A. Plant, William G. Blakeney

**Affiliations:** 1 Department of Orthopaedic Surgery, Royal Perth Hospital, Perth, WA 6009, Australia; kyle.raubenheimer@health.wa.gov.au (K.R.);; 2 Department of Emergency Medicine, Royal Perth Hospital, The University of Western Australia, Perth, WA 6009, Australia; daniel.fatovich@health.wa.gov.au; 3 Centre for Clinical Research in Emergency Medicine, Harry Perkins Institute of Medical Research, Perth, WA 6009, Australia; 4 School of Surgery, University of Western Australia, Perth, WA 6009, Australia

**Keywords:** e-scooter, orthopaedic, Australia, surgery, rideshare, emergency department, costs

## Abstract

The use of electric scooters (e-scooters) is increasing in Australia and internationally. The increasing availability of e-scooters has led to a rise in the number of injuries, with most patients sustaining orthopaedic injuries. This retrospective case series describes the incidence, management, and hospital costs of the orthopaedic injuries, which presented to the emergency department (ED) of the major trauma center in Western Australia. Data on demographics, ED dispatch destination, management, follow-up clinics, and hospital costs were collected between 2017 and 2022. Since June 2020, there have been 61 e-scooter crashes, which resulted in orthopaedic injuries, with more than half of the crashes occurring after the introduction of regional e-scooter sharing schemes. Thirty-two patients (52%) were admitted to the hospital. The most common orthopaedic fracture was to the upper limb (44%), followed by the lower limb (41%) and the axial skeleton (15%). Fourteen (23%) patients sustained more than one fracture. Twenty-two patients (36%) required operative management. The median number of outpatient clinic attendances per patient was 3 (interquartile range (IQR): 1–5), with inpatients requiring twice the number of clinics as compared to those discharged from the ED. The median cost per presentation was AU$5880.60 (IQR: AU$1283.10–AU$21,150.90) with inpatient costs exceeding those discharged from the ED. The range of the total costs was AU$413.80 to AU$100,239.80. The rise in the accessibility of e-scooters in Western Australia has led to a rise in ED presentations with orthopaedic injuries. Considering the recent implementation of e-scooter sharing schemes in metropolitan areas, ongoing surveillance of e-scooter injuries by clinicians and policy makers is warranted to inform harm minimization strategies.

## 1. Introduction

Marketed as a cost effective, time efficient, and environmentally friendly form of personal transportation, electric scooters (e-scooters) have gained widespread popularity since first appearing in 2017 [1]. In both metropolitan and regional centers of Western Australia (WA), an increasing number of independent companies introduced e-scooter sharing schemes, providing easy access to e-scooters for commuters and tourists [2,3]. In certain areas, e-scooters have gained significant momentum with traffic, increasing by 74% between 2019 and 2020 [4]. The increased availability of e-scooters through sharing schemes has been associated with increased e-scooter-related ambulance calls and emergency department (ED) presentations; this has been recognized across Australia and internationally [5,6,7,8,9,10,11,12,13,14]. 

The most common injuries sustained during helmeted e-scooter crashes are orthopaedic injuries [11,15]. A recent study from Victoria, Australia, noted that 81% of patients with e-scooter-related injuries who were admitted under orthopaedics required surgery [16]. Internationally, up to 30% of fractures require surgical fixation reflective of a novel mechanism of injury that is associated with increased orthopaedic workload and cost to the healthcare system [15,16,17,18,19,20,21,22,23,24,25,26,27,28,29]. Previous work from Australia has noted that between 12–25% of all ED presentations for e-scooter injuries are admitted to the hospital [5,6,9]. Preliminary financial data have indicated the costs associated with e-scooter presentations to the healthcare system in Australia, with ED presentations costing between AU$500 and AU$800 on average whilst the average cost for inpatient admissions have been calculated to be over AU$12,000 [6,9].

With the increasing use of e-scooters and continual introduction of trials across metropolitan areas, it is important to understand the harm associated with their use and the burden they place on the healthcare system. 

The primary aim of this study is to assess the incidence of patients attending the ED with e-scooter-related orthopaedic injuries, their dispatch destination, and management at a level one major trauma center in Australia. The secondary aims of this study included a description of patient demographics and to characterize the orthopaedic injury patterns. Hospital costs were calculated to establish the financial burden to the healthcare system. This study builds on previous work that utilized the trauma registry database, which captured admitted major trauma patients only and included all forms of injury [14].

## 2. Materials and Methods

### 2.1. Study Design

We performed a retrospective review of prospectively collected data using the ED Information System (EDIS). All e-scooter-related ED presentations to a major trauma center (Royal Perth Hospital) in Western Australia between 1 January 2017 and 14 June 2022 were identified.

### 2.2. Population

The EDIS was queried for all ED presentations that included the words “scooter”, “e-scooter”, and “electric scooter”. Two datasets were created with the first containing the words “e-scooter” or “electric scooter” with the second containing all remaining scooter presentations. The datasets were merged to include all e-scooter presentations. The data were manually reviewed (KS and KR) to remove inappropriate presentations that were not related to e-scooter crashes, such as injuries involving motorized scooters, electronic bikes (e-Bikes), unpowered scooters, pedal bicycles, electronic skateboards, wheelchairs, or other forms of wheeled transport. 

### 2.3. Inclusion Criteria

Inclusion criteria for the present study included:

Any ED presentation during the study period that involved an e-scooter, where the injuries were managed by the orthopaedic department.

Orthopaedic injuries included:

- Upper limb fractures and dislocations;
- Lower limb fractures and dislocations;
- Pelvic fractures;
- Spinal fractures.

Patients were identified using three techniques. First, ICD-10 codes were screened for fractures and dislocations. Second, the ED diagnoses were screened for inclusion of the words “fracture” and “dislocation”. Finally, all patients’ triage notes, clinical notes, and radiology reports were reviewed and assessed to see if a fracture or dislocation was noted during the ED presentation. If any of the 3 techniques were positive for fracture or dislocation, the patient was considered to have a fracture/dislocation. The investigator assessment served as a quality control step with misdiagnoses from the ICD-10 codes, ED diagnosis, or no radiological evidence of orthopaedic injury being excluded from the study. 

### 2.4. Exclusion Criteria

Exclusion criteria aimed to ensure relevance of the burden to orthopaedic services. Therefore, maxillofacial fractures, skull fractures, hand fractures, and rib fractures were excluded from the dataset, as these fractures are managed by the maxillofacial, neurosurgical, plastic and reconstructive surgical, and trauma surgical specialties, respectively. If a patient sustained any of the aforementioned fractures concurrently with orthopaedic injuries, only the non-orthopaedic injuries were excluded.

### 2.5. Outcomes

This study primarily aimed to determine the incidence, disposition, and management of patients who presented to the ED after having suffered orthopaedic injuries related to e-scooters. Outcomes included the number of presentations per year, disposition from the ED, and management (operative vs. non-operative). 

Secondary outcomes included injury patterns (according to anatomical region) and costs associated with orthopaedic injuries sustained following e-scooter use. All orthopaedic injuries that were sustained were counted in both isolated orthopaedic injuries and in patients with injuries involving other body systems (such as in polytrauma patients). Days to theatre and days to first orthopaedic clinic were calculated from time of hospital presentation. Number of outpatient appointments and “did-not-attend” clinics were recorded. For patients who met inclusion criteria, outcomes were obtained from the hospital information system, iSOFT Clinical Manager (i.CM) (DXC Technology, Sydney, Australia), which included management type, time to operation, and time to orthopaedic outpatient clinic. 

Hospital cost data were captured by the hospital Business Intelligence Unit (BIU). All patient presentations were linked by their hospital unit medical record number to the BIU database. Direct costs were defined as all measurable and identifiable activities involved in patient care. Staff salaries (including medical, nursing, allied health, and clerical) and treatment costs were included in the direct costs. Indirect costs are costs that are not directly related to patient activity but still provide some form of benefit to the patients. Examples of these costs include information technology, human resources, finance department, and executive director expenses. Radiology-specific costs were provided and included in the cost calculations. The BIU uses price weights for each patient and the National Efficient Price determined by the Independent Hospital Pricing Authority to determine the cost of health services. Cost calculations from index admission to last recorded outpatient appointment were collected between 1 January 2020 and 1 May 2023. Dates for hospital costs were determined by the first year that an orthopaedic e-scooter injury was recorded in the ED dataset, and up to the date at which the hospital cost calculations were requested to capture as many outpatient appointments as possible. Index admission was defined as the date of the emergency department presentation for e-scooter injury. 

From January 2020 to June 2021, ED costs were classified using the Urgency Related Group classification. From July 2021, ED presentations were classified and priced using the Australian Emergency Care Classification, which replaced the Urgency Related Group classification for activity-based funding purposes. The sum of direct costs, indirect costs, and radiology costs for a given ED presentation were defined as ED costs. Inpatient costs were classified according to the Australian Refined Diagnostic Related Group for activity-based funding purposes. Inpatient costs included the sum of direct and indirect costs per admission stratified by department, operation, and radiology costs. Outpatient costs were defined as direct and indirect costs billed to the orthopaedic department and radiology costs associated with outpatient appointments. Cost calculations included median cost with interquartile range (IQR) for ED costs, inpatient costs, outpatient costs, and total costs from index admission to last captured outpatient appointment. All costs were calculated in Australian dollars (AU$) in 2023. At the time of submission of this manuscript, the exchange rate of AU$ to United States dollars (US$) was 1 AUD to 0.64 US$ and 1 AU$ to 0.61 Euros. Outpatient “did-not-attend” appointments were determined by reviewing administrative costs associated with outpatient appointments that were not associated with a surgical team review billing.

All data were housed on a hospital-based information system that required 2-factor authentication for access with password encryption on all documents with patient datapoints. Once data were collected, all patients were de-identified prior to statistical analysis to protect patient confidentiality. This project received governance approval from the local Clinical Safety and Quality Unit (GEKO approval # 47431).

### 2.6. Statistical Analysis

Descriptive statistics were provided for the entire cohort and two subsets of patients based on ED disposition (hospital admission vs. discharged from the ED). All data were reviewed using histograms, and the Kolmogorov–Smirnov test was used to determine if data were normally distributed. For continuous variables, a Wilcoxon ranked-sum test was used to compare the 2 independent samples. For categorical variables, chi-square tests or Fisher Exact tests (if *n* < 5) were used to compare differences in demographics between admitted and discharged patients. Statistical significance was set at *p* < 0.05. Statistical analyses and visualizations were completed using R Statistical Package (R Foundation for Statistical Computing, Vienna, Austria) [30].

## 3. Results

**Figure 1:**
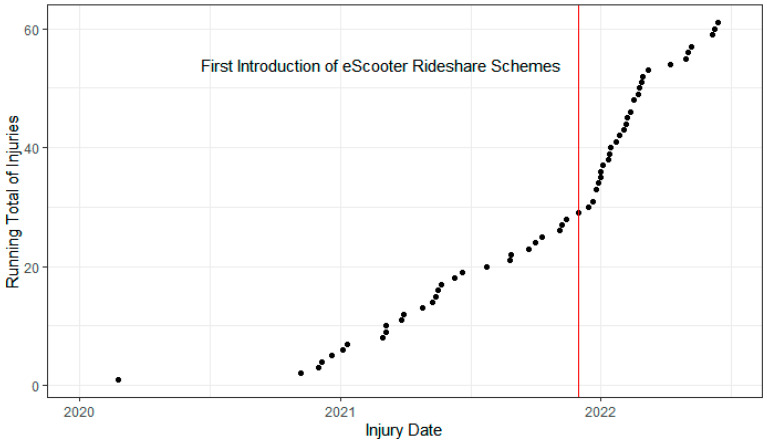
Running Total of Emergency Department Presentations e-Scooter-Related Orthopaedic Injuries Between January 2020 and June 2022. Red line indicates first introduction of e-scooter rideshare in Western Australia.

Between 1 January 2017 and 14 June 2022, 61 patients sustained orthopaedic injuries (Figure 1). There were no e-scooter crashes resulting in ED presentations with orthopaedic injuries between 1 January 2017 and 23 February 2020. No pedestrians hit by e-scooters were identified during the study period.

Table 1 details the demographics and administrative data of the cohort. The median length of stay for admitted patients was four days. Thirty-two patients (52%) were admitted to hospital for assessment and management of their injuries, and twenty-nine (48%) patients were discharged from the ED. Most patients were middle-aged males. There were no statistical differences for age or sex ratios between cohorts. In patients discharged from the ED, three patients attended the ED short stay unit overnight, and two patients returned for surgery both with a length of stay of one day. The median number of outpatient clinic appointments was higher for patients admitted to the hospital as compared to patients discharged from the ED. The median number of days to first booked outpatient appointment was nearly twice as many for admitted patients as compared to those discharged from the ED. Eight patients (28%) who were discharged from ED did not have an outpatient appointment booked as opposed to three (9%) admitted patients.

**Figure 2:**
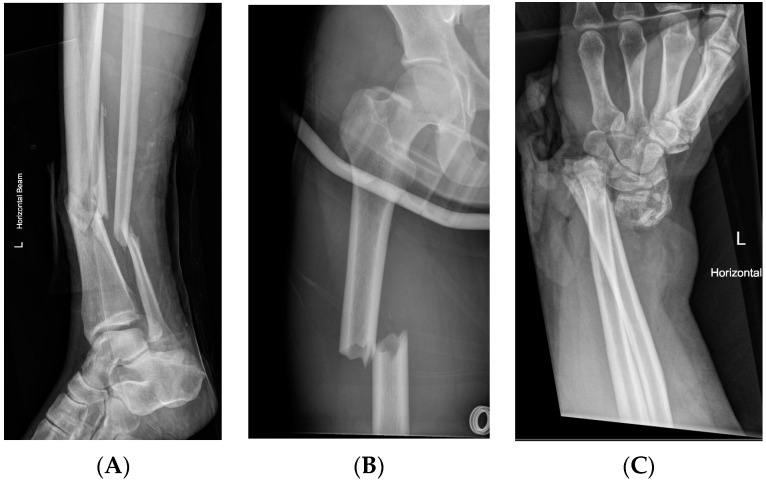
Plain films demonstrating high-energy nature of e-scooter-related orthopaedic injuries. (**A**) Open tibial and fibula diaphyseal fractures; (**B**) Midshaft femoral fracture; and (**C**) Open distal radius fracture.

Injury patterns are detailed in Table 2. A total of 75 injuries were identified. The most common orthopaedic fracture was to the upper, limb (44%), followed by the lower limb (41%) and the axial skeleton (15%). Examples of the high-energy nature of selected injuries sustained are demonstrated in Figure 2. Of the thirty-three upper limb fractures, twenty (61%) were radial bone fractures. Fourteen patients (23% of the total cohort) sustained more than one fracture and thirteen (93%) of these patients with multiple fractures were admitted.

**Figure 3:**
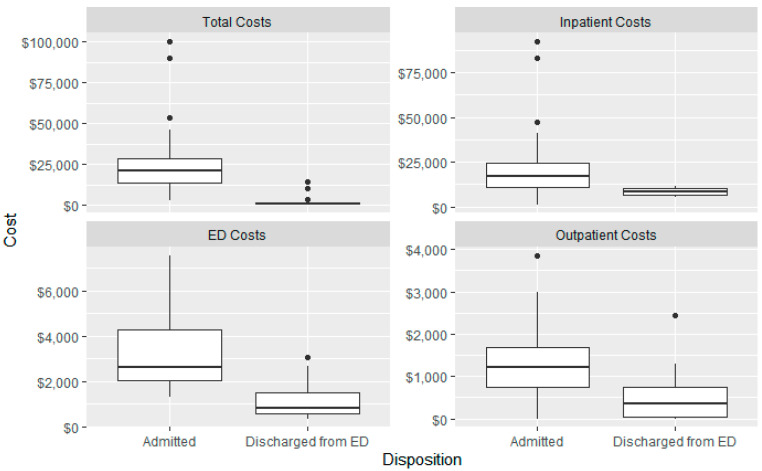
Comparison of Hospital Costs for Admitted Patients vs. Discharged from Emergency Department with e-Scooter-Related Orthopaedic Injuries.

All costs are reported in Table 3. The range of the total costs was AU$413.80 to AU$100,239.80. Boxplots illustrating the differences between patients admitted to the hospital and discharged from ED are detailed in Figure 3.

## 4. Discussion

This study has demonstrated a large increase in e-scooter-related ED presentations with orthopaedic injuries to the Western Australian state trauma center since 2020. In the present study, fifty-two percent of ED presentations with orthopaedic injuries were admitted. Observations regarding disposition from the ED highlight the healthcare burden imposed by e-scooter injuries. Whilst other studies have considered ED presentations of e-scooter-related orthopaedic injuries, this study considers disposition, follow-up, and the hospital costs (including outpatient appointments) from the index presentation.

The increase in ED presentations with orthopaedic injuries following e-scooter crashes may reflect the widespread introduction of e-scooters in the Western Australian community. The rise of e-scooter use has been driven by the public perception of their numerous benefits [31,32]: they are promoted as cost-effective, sustainable, and flexible on-demand travel solutions, particularly suited to urban environments [31,32]. The environmental benefits are further enhanced by the advent of ridesharing schemes, negating the need to own the vehicles and reducing both traffic congestion and vehicle emissions [32]. However, this new form of transport brings with it a new form of harm, particularly with regard to orthopaedic injuries [15]. Brisbane was the first city in Australia to introduce e-scooter share schemes in 2018 [9], with multiple cities and regional areas in WA following suit since December 2021 [33]. The City of Perth has recently commenced an e-scooter sharing scheme trial as of 18 March 2023 [34]. The introduction of e-scooter share schemes has been associated with increased incidence of e-scooter ED presentations and orthopaedic admissions across many parts of Australia [5,6,9,16]. These findings are supported by a United Kingdom study (London), which noted a threefold increase in orthopaedic injuries from e-scooter incidents following the introduction of a government e-scooter trial scheme [7]. Ongoing surveillance of e-scooter-related ED presentations in Western Australia is therefore warranted to inform harm reduction policies.

A proposed benefit of e-scooter usage is their environmental sustainability [2]. Due to their small size and portability, supporters of e-scooters propose that e-scooters are a viable solution to the “last mile” problem—the final part of a commute home between the end of public transport and a rider’s final destination [35]. The introduction of widely available rideshare e-scooters further reduce their environmental impact and accessibility [2,35]. Moreover, e-scooters are particularly suited to urban environments where traffic congestion is a concern and space is at a premium [2]. However, the injuries associated with e-scooter crashes may have a poorly considered environmental side effect in the resources required to treat orthopaedic injuries. In turn, the proposed environmental benefits of e-scooters may be at least partially offset by the burden placed on the environment through the provision of orthopaedic services [36]. To mitigate this environmental effect, orthopaedic services may consider implementing validated environmental sustainability assessments to reduce their impact on the environment [36]. These assessments and implementations are not e-scooter specific and are likely to provide benefits for all orthopaedic care at a given center. Further research contrasting the proposed environmental benefits of e-scooters offset with the environmental costs of treatment for injuries associated with e-scooter use is warranted.

Over half of the current cohort were treated and discharged from the ED, and over a third of all patients required surgery. Unsurprisingly, more admitted patients underwent surgery as compared to those discharged from the ED. This result is expected as more severely injured patients are more likely to be admitted than less severely injured patients. The differences in the current project as compared to other Australian studies is likely indicative of the orthopaedic focus of this project, whereas other studies considered all injuries [5,6,9,14]. The number of inpatients who underwent surgery is akin to those noted recently in Victoria, indicating an Australian trend for orthopaedic injuries [16]. 

Admitted patients had a significantly higher financial cost to the healthcare system for outpatient costs, ED costs, and total costs. Whilst ED costs were comparable to those seen in both Brisbane and the Northern Territory, inpatient costs were markedly higher in the present study as compared to those seen in the Northern Territory [6,9]. The reasons for this are unclear; however, it should be noted that their study investigated injuries of any type, and only six patients (11%) of their cohort were admitted [6]. Since the demographic profile of patients of the current cohort aligns with working age people, future studies may consider including return-to-work data to assess the cost to the economy. 

Injury patterns of this study were consistent with those found in the literature [11,15]. Short distance falls, combined with slow reaction times, were likely to result in upper limb fractures, particularly the radius and scaphoid [11,15]. Further, this study demonstrated that e-scooter use can be associated with high impact injuries, such as midshaft tibial and femur fractures, grossly displaced radius fractures, and pelvic fractures. 

We recognize limitations in the present study. First, the dataset used for the data captured was a combination of administrative data and clinical medical records, neither of which are designed for research. However, our three-tier fracture/dislocation identification strategy and dual-investigator manual record review minimizes the risk of missed injuries or incorrect patient inclusion. The present study focused on ED presentations and therefore did not include a direct measurement of injury severity. Whilst the length of hospital stay, hospital costs, and the type of injuries may give an indication of injury severity, we acknowledge that no direct measurement of injury severity was conducted in this study, and the results should be interpreted accordingly. The retrospective nature of this study did not account for the ownership type of e-scooter used (private vs. rideshare e-scooter). Whilst the results of the current study may suggest an association between the introduction of the first e-scooter rideshare program in Western Australia and an increase in the number of orthopaedic injuries sustained in e-scooter crashes, retrospective observational data cannot provide a causal link between these two factors. Furthermore, the state trauma center receives only adult patients (over 16 years of age). Pediatric patients who sustained e-scooter-related orthopaedic injuries are diverted to a nearby tertiary pediatric center. Finally, this was a single-center study. Future studies that include data from all tertiary centers within the state may provide a more holistic perspective of the orthopaedic burden of disease imposed by e-scooter use. Despite these limitations, this study provides a summary of the current orthopaedic burden posed by the emergence of a new form of transport. Taken together, the observations of ED disposition, management, and subsequent hospital costs summarize the orthopaedic burden on the Western Australian state trauma center posed by e-scooter-related orthopaedic trauma since 2017. 

## 5. Conclusions

Orthopaedic injuries associated with e-scooter usage are on the rise in Western Australia. Whilst many ED presentations were discharged, over half of the patients with e-scooter-associated orthopaedic injuries were admitted to the hospital. Admitted patients were more likely to undergo surgery and have higher ED, outpatient, and total costs. The introduction of e-scooter sharing schemes brings with them an increased risk of injury that policy makers and clinicians must be conscious of to reduce harm and financial costs to the community.

## Figures and Tables

**Table 1:** Demographics and Administrative Data in Patients with e-Scooter-Related Orthopaedic Injuries.

Variable	Total Cohort (*n* = 61)	Admitted (*n* = 32)	Discharged from ED (*n* = 29)	*p* Value
Age (yrs)	43 (33–53)	45 (37–52)	37 (33–55)	0.5
Sex (male: female)	49:12	25:7	24:5	0.88
LOS	1 (0–4)	4 (3–8)	-	Not applicable
Number of OPC appointments	3 (1–5)	4 (3–5)	2 (0–3)	<0.01
Days to first OPC	14 (10–21)	20 (14–23)	11 (9–14)	<0.01
Number of “did-not-attend” clinic	0 (0–1)	0 (0–1)	0 (0–1)	Not applicable
Surgery				
Yes	22/61 (36%)	20/32 (63%)	2/30 (7%)	<0.01
Days to surgery	2 (1–6)	2 (1–5)	9 (7–10)	Not assessed

All values are displayed as median, IQR unless otherwise stated. Days to surgery not statistically analyzed due to low sample size in the discharged from ED cohort. IQR—interquartile range; LOS—length of stay; OPC—outpatient clinic.

**Table 2:** Number of Patients with Orthopaedic Injuries Stratified by Operative vs. Non-operative Management.

Anatomical Location	Total Cohort (*n* = 61)	Operative Management (*n* = 22)	Non-Operative Management (*n* = 39)
Upper Limb Injuries	33		
Scapula	2	0	2
AC Joint Dislocation	1	0	1
Clavicle	6	1	5
Humerus	4	1	3
Radius	20	3	17
Lower Limb Injuries	31		
Pelvis	5	4	1
Neck of femur	1	1	0
Femoral shaft	3	3	0
Tibial plateau	4	4	0
Tibial shaft	8	5	3
Fibula	4	4	0
Patella	1	1	0
Ligamentous Knee Injury	2	2	0
Ankle	3	1	2
Spinal Injuries	11		
Cervical Spine	3	1	0
Thoracic Spine	5	1	4
Lumbar Spine	3	1	2

AC—acromioclavicular.

**Table 3:** Median Costs for e-Scooter-Related Orthopaedic Injuries.

Cost Type	Median Cost (IQR)
ED Costs	AU$1748.30 (AU$937.20–AU$2763.90)
Inpatient Costs	AU$16,236.00 (AU$8936.00–AU$24,099.00)
Outpatient Costs	AU$756.40 (AU$ 122.80–AU$1283.10)
Total Costs	AU$5880.60 (AU$1283.10–AU$21,150.90)

## Data Availability

The data presented in this study are available on request from the corresponding author. The data are not publicly available due to confidentiality.
